# Role of capping agents in the application of nanoparticles in biomedicine and environmental remediation: recent trends and future prospects

**DOI:** 10.1186/s12951-020-00704-4

**Published:** 2020-11-23

**Authors:** Rabia Javed, Muhammad Zia, Sania Naz, Samson O. Aisida, Noor ul Ain, Qiang Ao

**Affiliations:** 1 Department of Tissue Engineering, China Medical University, Shenyang, 110122 China; 2 Department of Biotechnology, Quaid-i-Azam University, Islamabad, Pakistan; 3 Department of Physics and Astronomy, University of Nigeria, Nsukka, 410001 Nigeria; 4 Institute of Regulatory Science for Medical Device, National Engineering Research Center for Biomaterials, Sichuan University, Chengdu, 610064 China

**Keywords:** Capping agents, Nanotechnology, Biomedicine, Environment, Nanoparticles

## Abstract

Capping agents are of utmost importance as stabilizers that inhibit the over-growth of nanoparticles and prevent their aggregation/coagulation in colloidal synthesis. The capping ligands stabilize the interface where nanoparticles interact with their medium of preparation. Specific structural features of nanoparticles are attributed to capping on their surface. These stabilizing agents play a key role in altering the biological activities and environmental perspective. Stearic effects of capping agents adsorbed on the surface of nanoparticles are responsible for such changing physico-chemical and biological characteristics. Firstly, this novel review article introduces few frequently used capping agents in the fabrication of nanoparticles. Next, recent advancements in biomedicine and environmental remediation approaches of capped nanoparticles have been elaborated. Lastly, future directions of the huge impact of capping agents on the biological environment have been summarized.
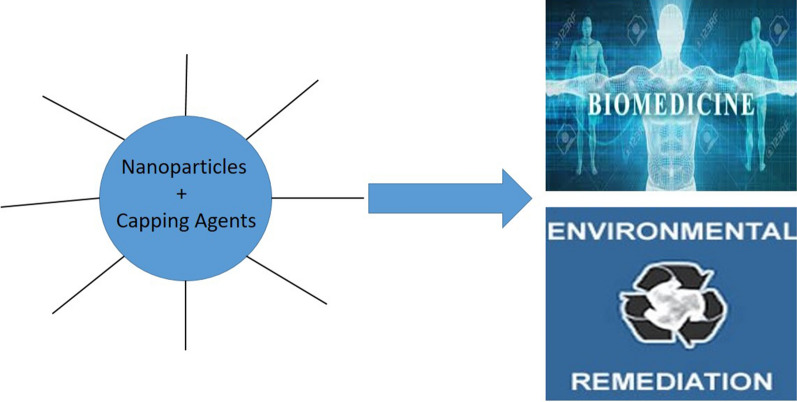

## Introduction

The particles ranging in size between 1 and 100 nm and possessing properties varying from the bulk material are defined as nanoparticles. In the last few decades, nanoparticles have been employed in various applied fields of science [1–3]. Usually, the nanoparticles are produced in large quantities on a commercial scale that are uncapped and having a larger size. These nanoparticles become hazardous to the environment when released in the form of large aggregates. Therefore, the choice of suitable capping moieties is key in stabilizing the colloidal solutions and their uptake into living cells and the environment. The surface chemistry and size distribution of nanoparticles get altered after capping with biocompatible surfactants [4–7]. Capping agents should be biodegradable, well-dispersed and biosoluble, biocompatible, and non-toxic in nature so that they can be easily utilized in the living system. Hence, their non-specific interaction with biological components reduces leading to alleviated cellular toxicity [8].

Evaluation of promising therapeutic potential and environmental influence of nanoparticles is of great interest to the researchers. The biological properties of nanoparticles are enhanced by surface capping. Capping agents are emerging therapeutic agents that show their clinical significance in synergy with the biocompatible nanoparticles to which they have been attached. The covalent bonding between the chains of capping ligands and the nanoparticles’ surface leads to steric hindrance providing ultimate stability to the nanocomposite. The percentage of atoms present on the surface increases at the nanoscale which gets further boosted by capping. Moreover, the agglomeration of nanoparticles gets minimized for a longer period of time employing appropriate capping agents [9].

Capping agents significantly modify the properties of colloidal suspensions of nanoparticles which makes them attractive candidates for biomedical applications such as drug delivery and theranostics in cancer. The biological reactivity and functionality with mitigated side-effects of nanocrystals are defined by their surface chemistry, morphology, and size which is attributed to suitable capping moieties. The small-sized nanoparticles have been explored in biomedicine and environmental remediation due to their novel characteristics obtained after surface tailoring [10, 11].

There are rising concerns regarding the safety and long-term toxicity of nanoparticles in the biological system that need to be resolved. Biocapping with aqueous plant extracts is an effective way of getting controlled growth of nanoparticles with minimum toxicity. However, since surface manipulation of nanocrystals is necessary to obtain monodisperse nanoparticles, polydispersion, and purification from by-products is a hurdle to the employment of this method on an industrial scale [12].

This review gives insight into the development of capped nanocomposites with ameliorated colloidal stability and biological functionalities. According to our knowledge, this is the first comprehensive report outlining the potential effects caused by the capped nanomaterials in the areas of biomedicine and environmental remediation.

### Capping agents in nanotechnology

**Fig. 1:**
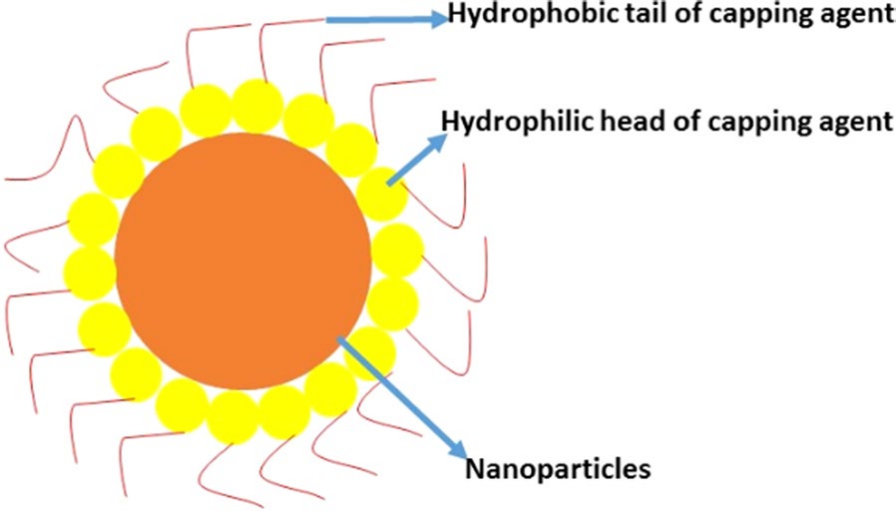
Nanoparticles covalently bound with capping agents

The frequent use of capping agents in colloidal dispersions to regulate nanoparticles controls the growth, agglomeration, and physico-chemical characteristics in a precise way  [13]. The capping agent is an amphiphilic molecule comprising of a polar head group and a non-polar hydrocarbon tail. Owing to the amphiphilic nature of capping agents, they confer the functionality and enhance the compatibility with another phase. The non-polar tail interacts with the encircling medium while the polar head interacts with the metal atom of the nanosystem [14] as shown in Fig. 1.

**Fig. 2:**
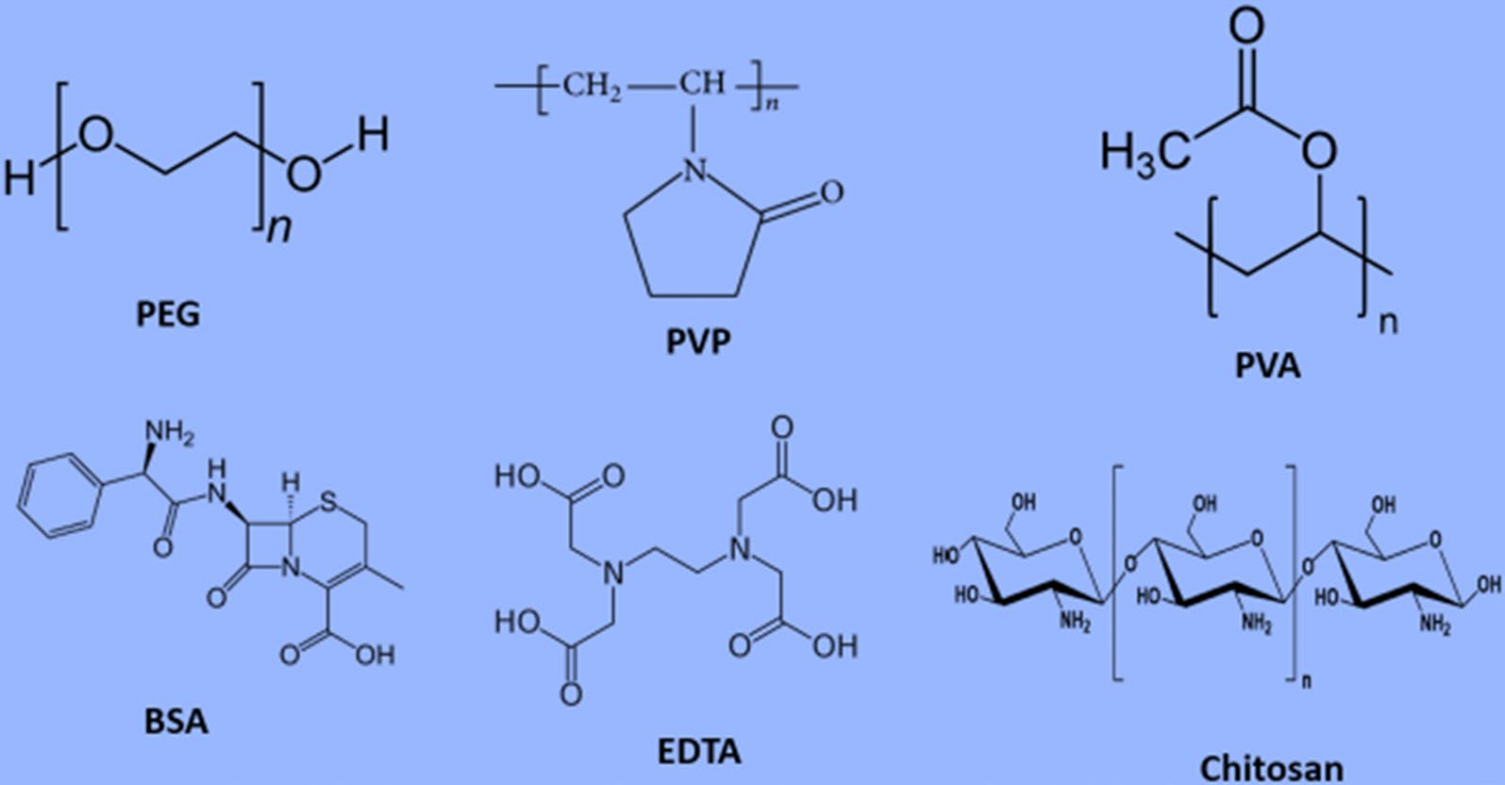
Different types of capping agents utilized in nanotechnology

Different types of capping agents have been used in nanoparticles’ synthesis including surfactants, small ligands, polymers, dendrimers, cyclodextrins, and polysaccharides. All of these have been successfully utilized as capping agents having the capability to induce subtle changes in nanoparticles elucidating tremendous therapeutic and environmental cleansing effect [15]. Figure 2 is representing the various types of capping agents.

### Polyethylene glycol (PEG)

PEG is a great biocompatible polymer with structural formula H–(O–CH_2_–CH_2_)_n_–OH. Synthesis of PEG takes place through the poly-condensation of ethylene glycol in the presence of a catalyst either acidic or basic, producing a lower molecular weight product. It is soluble in both aqueous and organic solvents. This feature enhances both its biocompatibility and processability. It is less toxic and non-immunogenic. Hence, coupled as a surface modifier with other compounds including biomaterials, micelles, and particles for active molecule transport, and also for physical and chemical hydrogels. In vivo, PEG is not detached hydrolytically. Nevertheless, its hydrophilic properties grant a greater water affinity and biodegradability to the polymer [16].

PEG has been used as a capping agent in nanotechnology extensively for several purposes such as the biomedical field for sustained and targeted drug delivery. The coating of metal and metal oxide nanoparticles with PEG takes place in wet-chemical synthesis [17]. PEG has been reported in various studies as a capping agent for metal nanoparticles involving gold (Au) [18], silver (Ag) [19], zinc (Zn) [20] to reduce cytotoxicity, improve stability, and biocompatibility associated with metal nanoparticles.

### Polyvinylpyrrolidone (PVP)

PVP, also known as povidone or polyvidone, is a polymer (C_6_H_9_NO)_n_ soluble in water. PVP is composed of polymerization of monomer *N*-vinylpyrrolidone. It is light, flaky, and hygroscopic powder. It absorbs approximately 40% of water by its mass. It consists of outstanding moistening properties. Hence, it makes films forming a compelling coating agent. PVP with its exclusive physico-chemical properties like solubility both in water and organic solvents, biocompatibility, chemical stability and non-toxicity makes it a potential biomaterial in many considerable medical and non-medical purposes. PVP is extensively used in various medical products, cosmetics, and haircare products. The popular uses of PVP in a pharmaceutical industry include manufacturing of drugs as a common ingredient in tablets, granules, pellets, soft gelatine capsules, gels, hydrogels, films and coatings, membranes and mats of nanofibers, powders, syrups, oral or injectable solutions, coatings for medical devices, contact lenses and many others [21, 22].

PVP is one of the significant capping agents that have been utilized in nanotechnology to overcome drawbacks associated with conventional methods of preparation of nanoparticles such as their toxicity, size, and agglomeration. Hence, ecofriendly nanoformulations are obtained using PVP having more applicability [23, 24]. In various researches, PVP has been employed as a capping agent around metal nanoparticles such as Iron (Fe), silver (Ag), gold (Au), zinc (Zn), etc. [25].

### Polyvinyl alcohol (PVA)

PVA is one of the high performance capping agents used in nanotechnology [26, 27]. PVA, which is essentially made through hydrolysis of polyvinyl acetate, is a synthetic polymer owning great hydrophilicity, biocompatibility, and biodegradability. PVA has been used as a stabilizing agent, for shape and size control of Ag nanoparticles to protect against water [28]. PVA is used in various biomedical arenas. PVA hydrogels hold considerable potential for their usage in tissue engineering and artificial grafts [29]. To improve the optical emission, crystallinity, and size dispersion, ZnO nanoparticles capped with PVA were prepared by the sol–gel method [30]. Iron oxide or magnetite nanoparticles are most likely to agglomerate due to magnetic forces, and it compromises the effectiveness and potential use of these nanoparticles. Therefore, PVA has been employed as a capping agent and glutaraldehyde as a cross-linker in the fabrication of iron oxide nanoparticles. This has not only prevented the agglomeration but also the oxidation of iron oxide nanoparticles via a two-step method in which first the iron oxide nanoparticles were prepared, and then coated and crosslinked with PVA and glutaraldehyde [31].

### Bovine serum albumin (BSA)

One of the most abundant and well-characterized ubiquitous proteins present in the plasma of mammals is serum albumin (SA). The key role of this protein is to maintain the level of pH in the blood, regulating the colloidal osmotic pressure and transporting different substances of various natures such as ionic, hydrophilic, and hydrophobic. The molecular weight of bovine serum albumin (BSA) is ~ 66 kDa and consists of 582 amino acids, with 35 threonine and 32 serine amino acids. Due to the presence of charged functional groups, including carboxyl, sulfhydryl, and amino, BSA finds numerous binding sites. These sites also assist in the binding of different therapeutic systems like drugs, poly-conjugated dyes, and nanoparticles. As a capping agent, BSA increases the bioavailability of loaded nanoparticles. It is taken up by cells through certain receptors present on the surface of tumor cells as a source of nutrient and amino acid. The mild reducing property of BSA is due to the hydroxyl group, just like PVP, capable of fabricating metal nano-plates, for instance, palladium (Pd), gold (Au), platinum (Pt), and silver (Ag) [32, 33].

Jyothi Kumar et al. (2019) reported the stabilization of Au nanoparticles by using BSA as a capping agent. BSA has exhibited excellent stabilizing as well as size control properties in bio-conjugated Au nanoparticles [34]. In another study, a sensor was developed for the detection of heparin and protamine using Au nanoparticles and BSA capped cadmium sulfide quantum dots (CdS QDs) based on the inner filter effect [35]. BSA also acts as a structure-directing agent as well as controlling the assembly, nucleation, and growth of nanoparticles. Cuprous oxide (Cu_2_O) nanoparticles capped with BSA have been reported to produce a hierarchical structure emulating biomineralization via the facile method [36].

### Ethylene diamine tetra acetic acid (EDTA)

EDTA is a water-soluble polymer commonly used as a chelating agent to dissociate cells from the extracellular matrix (ECM) by binding to divalent metal ions. Also, EDTA has been utilized as a complexing agent for the removal of metal ions [37]. EDTA has gained significant importance in nanoscience, being used as a stabilizer in the fabrication of nanoparticles. It efficiently controls the morphology and size of nanoparticles. It has been used as a capping agent in the preparation of various metal nanoparticles including gold (Au), zinc (Zn), copper (Cu), chromium (Cr), and cadmium (Cd) [38, 39]. Rahal et al. [40] synthesized nickel oxide (NiO) nanoparticles capped with EDTA via co-precipitation method and results showed that EDTA capped nanoparticles were smaller in size as compared to the pure NiO nanoparticles with enhanced surface magnetization properties. EDTA was also employed in the synthesis of magnetic iron oxide nanoparticles to enhance the mono dispersion via high-temperature hydrolysis reaction resulting in more water solubility and enhanced magnetic properties [41].

### Chitosan

Chitosan is a co-polymer comprising d-glucosamine and *N*-acetyl-d-glucosamine produced via alkaline deacetylation of chitin naturally occurring in the crustaceans or hydrolysis of chitin by the enzymatic action of deacetylase. After cellulose, this is the second most abundant biomaterial [42]. Although chitin is not soluble in dilute acids, chitosan is an acid-soluble polymer [43]. Lately, chitosan due to its exceptional biological properties has drawn substantial consideration in the biomedical field. In terms of biological applications, its fascinating properties include biocompatibility, biodegradability, non-toxicity, anti-carcinogenicity, immune-enhancing, and antimicrobial activity. It degrades both in vitro and in vivo into small monomers without producing any adverse effects [44]. In nanoscience, chitosan has been extensively used in the preparation of metal nanoparticles as a stabilizer and promoting wet-chemical synthesis, although the bonding between chitosan and metallic nanoparticles is still a debate. For instance, the linkage between platinum (Pt) nanoparticles and chitosan has been suggested to be via amino and secondary hydroxyl groups [45].

Chitosan has been used as a capping agent to stabilize the colloidal dispersion, and to control the morphology and optical properties of metal nanoparticles including gold (Au) [46], silver (Ag) [47], copper (Cu) [48], etc. Capping of Au nanoparticles with chitosan is used as a temperature manipulation sign. Au nanoparticles transform its color and intensity with variation in storage temperature and its duration. This difference in color and intensity is principally owing to the change in shape and size of chitosan capped Au nanoparticles, potentially used for the development of sensors in food [49].

### Plant extracts

**Fig. 3:**
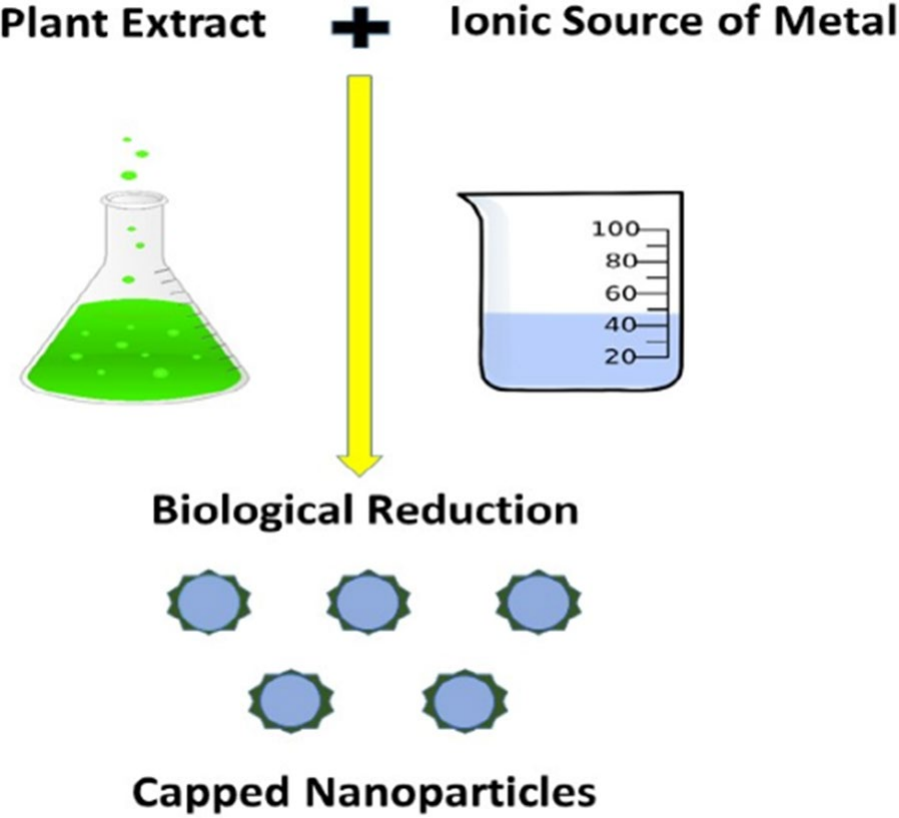
Schematic representation of the green synthesis of nanoparticles

Fabrication of nanoparticles using extracts from plants as potential capping and reducing agents is often known as green synthesis. The extracts from leaves, fruit, roots, and seeds of the plants can influence the properties of the formulated nanoparticles by enhancing their functionality optimally for biomedical applications [50–52]. Much work has been conducted using a green synthesis approach to tailor the properties of nanoparticles. Figure 3 illustrates the process of green synthesis of nanoparticles.

Anddresa et al. [53] used *Virola oleifera* exudate as a potential capping and stabilizing agent for the formulation of gold (Au) nanoparticles. With the capping agent, the zeta potential of the formulated Au nanoparticles was observed to shift from a negatively charged to a positively charged surface. The capped Au nanoparticles showed a low toxicity effect and high antioxidant activity. Rashid et al. [54] used four different plants (*Rumex dantatus*, *Bergenia ciliata, Rumex hastatus, and Bergenia stracheyi*) as potential capping agents to formulate silver (Ag) nanoparticles. The different formulated Ag nanoparticles showed strong antibacterial activity against six bacterial strains. The activity of the formulated nanoparticles was increased with the rise of their concentration. Moreover, an eco-friendly synthesis protocol using an extract of the bark of *Solanum trilobatum* as a potential capping agent for the formation of Ag nanoparticles was demonstrated by Ramanathan et al. [55]. The *S. trilobatum* capped Ag nanoparticles showed an enhanced antimicrobial and antifungal activity against *E. coli*, bacillus species, and *Aspergillus niger.* Madubuonu et al. [56] used two different extracts of *Psidium guajava* and *Moringa oleifera* for the preparation of iron oxide (FeO) nanoparticles for antibacterial and photocatalytic activity. From their observation, the composite extract gave a better activity than the individual extracts by providing mutual stability to FeO nanoparticles.

Angel et al. [57] prepared nickel oxide (NiO) nanorod via *Phoenix dactylifera* as a potential reducing and capping agent. The formulated nanorod showed strong toxicity over A549 cells, and good antibacterial and photocatalytic activity. Similar results were observed by Aisida et al. [51], they synthesized iron (Fe) nanorod using the green extract of *Moringa oleifera* as a potential reducing agent for antibacterial activity against six human pathogenic strains. They observed a strong susceptibility of the pathogens against Fe nanorods. Tahir et al. [58] used *Artemisia vulgaris* (AV) leaves extract as a potential reducing agent to synthesize Ag nanoparticles. The obtained AV-Ag nanoparticles showed strong antibacterial activity. The antioxidant and the cytotoxicity effect of AV-Ag nanoparticles showed a promising potential against MCF-7 and HeLa cell lines. A similar report was published by Nithya et al. [59] using ionic liquids from *Justicia adhatoda* extract as a templating and stabilizing agent to enhance the surface morphology of three different precursors; Au, Ag, ZnO nanoparticles and their composites. The composite samples exhibited strong antibacterial and anticancer activity against *E. coli, Staphylococcus aureus*, and HeLa cancer cells. Senthilkumar et al. [60] reported the synthesis of zinc oxide (ZnO) nanoparticles capped with *Tectona grandis* leaves extract for antiarthritic, anticancer, antioxidant, antibacterial activity, and in vitro cytotoxicity. The obtained results enhanced the antibacterial activity against both the Gram-positive and Gram-negative bacterial strains. The sample also showed enhanced denaturation of protein as well as proteinase activity at 200 μg ml^−1^ dosage. The anticancer activity was also tested against osteoblast MC3t3-E1 cell lines with a profound reduction in the size of the tumor.

Anticancer, antibacterial, and antileishmanial activity of green synthesized hematite were observed by Naz et al. [61]. They used *Rhus* *punjabensis* extract as a potential reducing agent to form hematite nanoparticles. The formulated nanoparticles also showed significant cytotoxic effect against HL-60 leukemic and DU-145 prostate cancer cell lines with ED_50_ values of 11.9 and 12.79 μg/ml, respectively. Aqueous extract of *Hygrophila spinose* was used to synthesize Au nanoparticles by Swaha et al. [62]. The formulated nanoparticles showed a small particle size with the help of the capping agent and exhibited potential anticancer activity against ovarian, breast, and brain cancer cell lines. Antioxidant and free radical scavenging activities of Ag nanoparticles prepared from the extract of *Piper longum* were presented by Renuka et al. [63]. The anticancer activity conducted against HeLa cells (cervical cancer cell line) showed non-toxicity of the fabricated Ag nanoparticles and gave a maximum IC50 value. Besides, antilarvicidal activity of the nanoparticles with efficient mortality was obtained against *Anopheles stephensi*, *Aedes aegypti* and *Quinque fasciatus* having LC_50_ and LC_90_ values of 8.969 ppm and 16.102 ppm, 14.791 ppm and 28.526 ppm, and 18.662 ppm and 40.903 ppm, respectively after 72 h of exposure.

### Influence of capped nanoparticles in biomedicine

Capping or stabilizing agents are essential in the fabrication of nanoparticles to enhance their biomedical functionality by reducing their toxicity and enhancing their biocompatibility and bioavailability in living cells. They prevent clusters or aggregates of nanoparticles, enhance their colloidal stability, and prevent uncontrolled growth of nanoparticles (especially the metal and metal oxide nanoparticles). The different types of capping agents also determine the particle size and morphology, and their magnetic, optical, and catalytic properties. For biomedical applications such as drug delivery in cancer therapy [64–66] and antimicrobial activities, [51, 56, 67], the bio-conjugate of the nanoparticles is very important. This involves the use of biocompatible, non-toxic, and biodegradable moieties as potential capping agents. Table 1 provides an overview of a few capped nanoparticles and their applications in the biomedical field.

**Table 1:** The nanoparticles with their capping agents conferring them with different biological activities

Capping agents	Nanoparticles	Biological activity	References
Chitosan	Liposomes	Antidiabetic	[68]
*Parkia speciosa* extract	Ag	Antioxidant	[69]
PVA	Ag	Anticancer	[70]
PEG	TiO_2_	Anticancer	[71]
Chitosan	ZnO	Antibacterial, antioxidant, antidiabetic, cytotoxic	[72]
Chitosan	FeO	Antibacterial and antioxidant	[73]
Chitosan + PEG + PVP	Fe_3_O_4_	Anticancer drug delivery	[74]
PVP	WO_3_	Anticancer	[75]
PVA	Mg	Anticancer	[76]
PVA/Guar Gum	Fe_3_O_4_	Anticancer	[77]
PVA	Cu	Antibacterial	[78]
*Aloe vera* extract	Fe_3_O_4_	Anticancer	[79]
Cellulose	Ag	Antibacterial	[80]
Alginate	ZnO	Antibacterial	[81]
Ethylene glycol	ZnO	Antifungal	[82]
Dodecanethiol	Ag	Antifungal	[83]

**Fig. 4:**
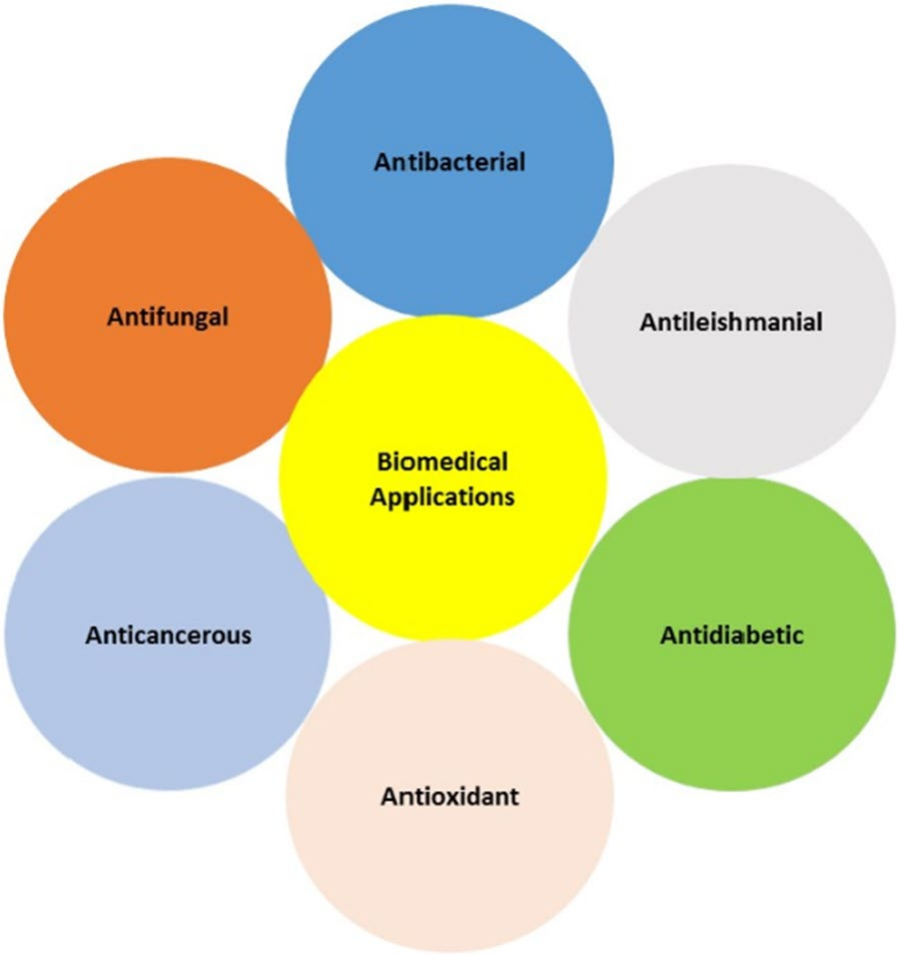
Capping agents of nanoparticles affecting different areas of biomedicine

The capped nanoparticles have been extensively studied for their applications in biomedicine including antimicrobial, antioxidant, anticancer, and antidiabetic activities as shown in Fig. 4.

### Antimicrobial activities: antibacterial, antifungal, antileishmanial

Microorganisms are the major culprit of infectious ailments throughout the globe. Scientists from all around the world are trying to fight against developing microbial infections. For instance, generally used conventional drugs are incompetent to show their effectiveness against microbial infections. The most common factor behind this is the overuse of these drugs, hence microbes develop resistance against drugs [84]. Moreover, some antimicrobial drugs are particularly toxic and irritant so there is great attention in searching novel ways to formulate safe and cost-effective antimicrobial agents. Currently, prevention and inhibition of microbial infections have been the most active research area in healthcare. This urges the growth and development of an effective and novel materials with superior antimicrobial properties. A good substitute for this purpose is nanotechnology [85]. Former reports have shown that antimicrobial preparations in the form of nanoparticles could be employed as an effective antibacterial agent [86]. Plenty of development has been done in the pharmaceutical industry using monodispersed nanoparticles to treat antimicrobial ailments. Various types of nanomaterials are in practice as an antimicrobial agent. However, the fabrication of nanoparticles with the desired shape and size is still challenging [87]. Hence, the synthesis, characterization, surface decoration, and functionalization of nanomaterials decipher the promise of developing new generation antimicrobial (bactericidal) materials [88].

Due to the instability (agglomeration), toxicity, size, and shape defects, nanomaterials are generally surface modified to enhance their antimicrobial properties. Literature supports the positive effects of surface modification on the antimicrobial potential of nanoparticles. For example, chitosan functionalized silver (Ag) nanoparticles exhibited superior antibacterial activity due to higher solubility and release of Ag^+1^ ions [89]. Spherical Ag nanoparticles functionalized with core–shell magnetic chitosan microspore demonstrated efficient antimicrobial activity and act as smart antifouling agents [90]. Ag nanoparticles coated with PVA/aminopropyltriethoxysilane revealed superior fungicidal activity [91]. Moreover, the capping of Ag nanoparticles with PVP and glucantime (antimicrobial drug) produced promising results against *Leishmania amazonensis* [92]. In another study, the PVP-360 capped Ag nanoparticles demonstrated obvious improvements in the antibacterial activities [93]. This was ascribed due to the effective prevention of agglomeration that resulted in the efficient stabilization of nanoparticles. The PVP and PEG-modified Ag nanoparticles [94], ZnO nanoparticles, and CuO nanoparticles [9, 95] exhibited pronounced antibacterial activities. Moreover, the functionalization of Ag nanoparticles with EDTA, PEG, PVP, and PVA was tested for antimicrobial potential [96]. Results showed that PVP coated Ag nanoparticles possess superior antimicrobial activity due to their smaller size as compared to other coated nanoparticles. Literature also stated that comparatively small particles demonstrate relatively higher antibacterial activity [97]. Besides this, ZnO nanoparticles capped with chitosan elucidated significant antimicrobial activity against fungus (*Candida albicans*) and bacterial strains (*Micrococcus luteus* and *Staphylococcus aureus*). Moreover, ZnO–Chitosan nanoparticles also displayed marked biofilm inhibition activity against *M. luteus* and *S. aureus* [98]. Chitosan functionalized gold (Au) nanoparticles were effective against the bacterial endotoxin lipopolysaccharide (LPS) [99]. From these findings, we could say that antimicrobial activities of nanoparticles are exclusively dependent on their physical attributes, i.e., adsorption of nanoparticles to the cell wall and their interaction, and denaturation of cell wall proteins which subsequently lead to the production of porous structures and marked alterations in the structure of cell membrane. This eventually enhances the permeability of the cell membrane and allow the movement of extracellular fluids inside the cell. Precisely, Ag nanoparticles impair the cell membrane via electrostatic attractions and effect certain membranous enzymes, damages the proton motive force (PMF), and finally cause cell lysis [100, 101]. From the literature discussed above, we can conclude that the functionalization of nanomaterials is crucial in the enhancement of their antimicrobial properties and useful in treating infectious diseases.

**Fig. 5:**
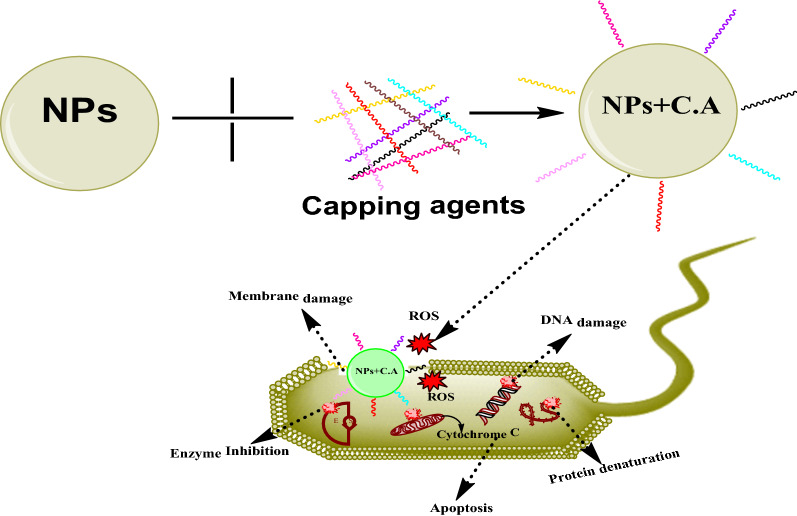
Proposed mechanism of antibacterial action of capped/modified nanoparticles

Figure 5 explains the mechanism of antibacterial action of capped nanoparticles.

### Antioxidant activities

Antioxidants are compounds produced naturally to defend the human body from the side effects of free radicals. Antioxidants scavenge the free radicals that produce reactive oxygen species (ROS), like nitric oxide (NO), hydroxyl radical (OH^−^), and superoxide anion (O_2_^−^) in healthy organisms as a defense system. Accumulation of ROS leads to varied chronic pathologies, for example, the development of cancer, cardiovascular, and neurodegenerative illnesses. Natural antioxidants are obtained from vegetables like quercetin. Quercetin (3,5,7,3′-4′-pentahydroxy flavone) is one of the flavonoids consisting of significant pharmacological activities including antioxidant potential. Despite its advantages, its clinical use is compromised due to low bioavailability and oral absorption due to less solubility in water. Therefore, gold (Au) nanoparticles are surface-functionalized with the capping of quercetin to enhance the bioavailability of the compound, hence the antioxidant activity. Nitric oxide (NO), 2, 2-diphenyl-1-picrylhydrazyl (DPPH) and 2,2′-azino-bis(3-ethylbenzothiazoline-6-sulfonic acid) (ABTS) free radical scavenging assays were performed to evaluate the antioxidant activity of quercetin capped Au nanoparticles. Results demonstrated that capped Au nanoparticles exhibited higher antioxidant activity as compared to quercetin alone [102].

Metal oxide nanoparticles exhibit great antioxidant potential but the use of uncapped nanoparticles is hindered due to their toxicity. For example, CuO nanoparticles capped with PEG and PVP were synthesized by co-precipitation method and antioxidant assays were carried out including antioxidation assay, reducing power assay, and DPPH-free radical scavenging assay. Both CuO-PEG and CuO-PVP nanoparticles exhibited excellent antioxidant, reducing power and DPPH-free radical scavenging activity. Most interestingly, the antioxidant activities were higher in capped nanoparticles as compared to uncapped ones [95, 102]. Similar results were obtained from another study in which ZnO nanoparticles were capped with PEG and PVP showing higher antioxidant activity by capped nanoparticles as compared to uncapped nanoparticles. This can be attributed to the alteration in the surface chemistry, morphology, and size of nanoparticles which substantially contribute towards the improvement of the biological properties of nanoparticles [9]. Antioxidant capacities of chitosan capped selenium (Se) nanoparticles have also been studied. Se was stabilized to its zero state via chitosan capping to harness its full potential of an antioxidant agent by increasing bioavailability and lowering the toxicity. Lipid peroxide, DPPH, and ABTS assays revealed that chitosan capped Se nanoparticles scavenge free radicals at diverse levels. A month-old chitosan capped Se nanoparticles apprehended such a higher ABTS scavenging ability that the value could reach up to 89.44 ± 5.03%. In the cell culture assays using BABLC-3T3, the accumulation of the intracellular ROS could be inhibited by chitosan capped Se nanoparticles with better penetration and lower toxicity [103].

### Anticancer activities

**Fig. 6:**
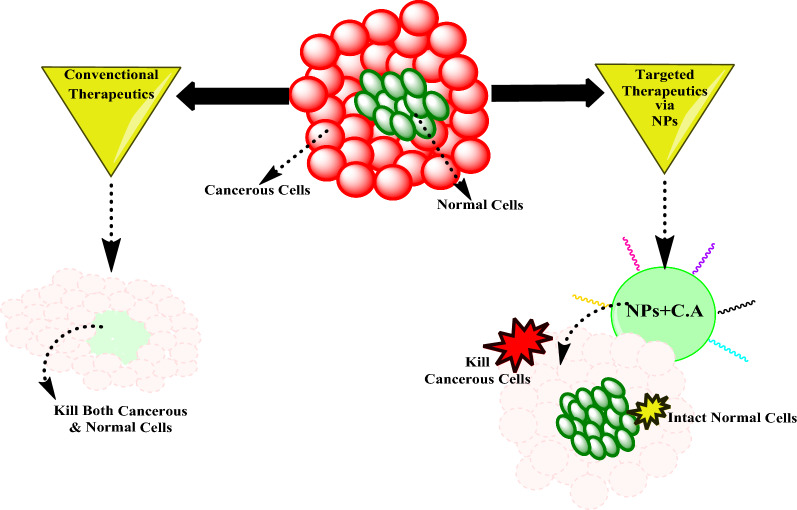
Comparison of mode of anticancer action of conventional therapeutics versus capped/modified nanoparticles

Despite the presence of conventional cancer treatments including radiotherapy, chemotherapy, or surgical removal, there are many side effects such as hair loss, pain, nausea, vomiting, and damage to normal cells. Nanotechnology is an alternative approach to develop combinational chemo-immunotherapies against different types of cancers. The goal of incorporating nanoparticles in treating cancer is to provide targeted drug delivery to overcome the side effects of conventional therapies (Fig. 6). Although nanoparticles exhibit tremendous tumor and cancer-killing properties, they cannot be applied clinically due to their toxicity. Hence, capped nanoparticles are utilized to treat different types of cancer such as breast, colorectal, liver, ovarian, and lung cancer [104].

Nanoparticles act as potential drug delivery systems because they escalate the retention time of the drug in the blood, lower the efflux, and undergo targeted delivery. Methotrexate (MTX) is an anti-cancer drug that can cause renal and hepatic toxicity at higher administration doses. Methotrexate silver nanoparticles (Ag-MTX) capped with PEG were synthesized via chemical reduction method. Biocompatibility and anticancer activity were evaluated. PEG capped Ag-MTX nanoparticles displayed increased anticancer activity against the MCF-7 cell line. Also, the hemolytic activity of nanoparticles was significantly reduced as compared to MTX administered alone. Therefore, PEG-Ag-MTX nanoparticles were proved to be the prospective nano-carriers of methotrexate with reduced side effects [105]. Moreover, magnesium (Mg) nanoparticles coated with PEG act as a drug delivery vehicle for anticancer drug, 2-Methoxyestradiol (2ME). These drug carrier nanoparticles were tested against prostate cancer cell lines and results revealed the decrease in tumor activity of cells, and this drug carrier system can be used to treat prostate cancer [106].

Magnetic (Fe_3_O_4_) nanoparticles are promising candidates for drug delivery as they are biocompatible and can be directed under an external magnetic field for magneto-therapy. Fe_3_O_4_ nanoparticles have been used as a drug carrier for various anticancer drugs such as doxorubicin, daunorubicin, 5-bromotetrandrine, and anti-HER2 immunoliposomes to assess their therapeutic potential in breast cancer. Dual paclitaxel (PTX)/superparamagnetic iron oxide (SPIO)-loaded poly (lactic-co-glycolic acid) (PLGA)-based nanoparticles were developed for cancer therapy. These nanocarriers showed effective antitumor activity in vitro against the CT26 cell line. These nano-entities have not only antitumor characteristics but also imaging properties, therefore, concurrently achieving multifuctionalities including targeting, imaging, drug delivery, and real-time monitoring of therapeutic response [107].

### Antidiabetic activities

Diabetes is a metabolic disorder characterized by elevated levels of glucose in the blood. It has affected more than 100 million people worldwide. Consequently, there is a requirement to develop medicines at nanoscale inhibiting the levels of carbohydrate-hydrolyzing enzymes with a greater extent of specificity and achieving the utmost therapeutic effectiveness with negligible side- effects. Antidiabetic activity of PEG and PVP coated CuO nanoparticles was carried out using the α-amylase inhibition assay. Capped CuO nanoparticles showed higher inhibition, i.e., CuO-PEG and CuO-PVP nanoparticles revealed 32.23% and 37.75%, respectively as compared to uncapped CuO nanoparticles achieving only 19.17% of enzymatic inhibition. It is due to a decrease in size and more surface area owing to the major movement of atoms towards the outer surface of capped nanoparticles [95]. Also, the antidiabetic assay against PEG and PVP capped ZnO nanoparticles declared efficient antidiabetic activity in comparison to uncapped ZnO nanoparticles [9].

Folic acid-functionalized chitosan nanoparticles were synthesized by the ionotropic gelation method. The effects of these nanoparticles on stability, oral bioavailability, and hypoglycemic activity were observed following oral administration in vivo. Formulated nanoparticles demonstrated the persistent release of insulin up to 24 h. Encapsulated insulin remained stable both conformationally and chemically. Uptake studies in Caco-2 cell lines deduced remarkably higher effects, so was the bioavailability through oral administration [108]. Chitosan-EDTA coated (CEC) liposomes encapsulating insulin were synthesized by reverse-phase evaporation method and the shape obtained was either spherical or ellipsoidal. The hypoglycemic effect was evaluated by the oral administration of nanoparticle formulations in mice. Proteolytic activity of pepsin and trypsin was repressed by CEC-coated liposomes that enhanced the enteral absorption of insulin [109].

### Impact of capped nanoparticles in environmental remediation

Environmental pollution increases day by day and now it becomes one of the most serious global menace facing by society as it produces irreversible damage. Continuous urbanization and the breakneck leap of industrialization have disturbed the balance of environmental composition through the release of hazardous materials, smoke, and noxious gases which consequently lead to the toxic effects on living things. Furthermore, over usage of natural resources due to overpopulation, a large number of vehicles, and higher release of smoke from industry and many other factors lead to nature’s destruction [110, 111]. Examples of some hazardous materials include heavy metals, pesticides, herbicides, fertilizers, residual pharmaceutical products, poisonous gases, effluents generated by the industries, sulfur-containing compounds, oil spills, matter particulates, sewage, pathogens, organic compounds, etc. These materials release into the environment and eventually contaminate the soil, water, and air, hence compromising the ecosystem and health (like Alzheimer’s, dementia, or deafness) [110, 112]. Currently, under present circumstances, sustaining the pure and healthy air and water environment is a key challenge.

Fortunately, novel technologies are now in practice to remediate undesirable products from the air, water, and soil [113]. Among them, nanotechnology exhibits great potential to build and use novel and cost-effective techniques for the detection and monitoring of contaminants, degradation via catalysis, adsorptive removal, and cleaning of the environmental pollutants [113]. In comparison to the bulk materials, nanotechnological products have novel physical and chemical attributes due to their smaller size (< 100 nm) [114, 115] that results in a higher surface to volume ratio which makes them efficient catalyst. Furthermore, higher interfacial reactivity and specific functionalization make nanoproducts as newly miniaturized, precise, and sensitive nanosensors for targeted detection and remediation of undesirable materials. Recently, a myriad of nanomaterials such as carbon nanotubes, polymers, dendrimers, metallic oxide nanoparticles, and many more have been synthesized and employed for the purification of water, air, and soil.

**Fig. 7:**
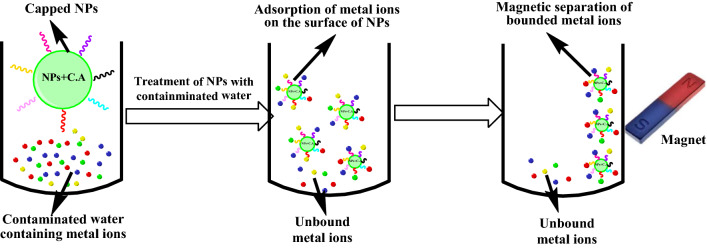
Magnetic separation of metal ions from the contaminated water by the use of capped/modified nanoparticles

**Fig. 8:**
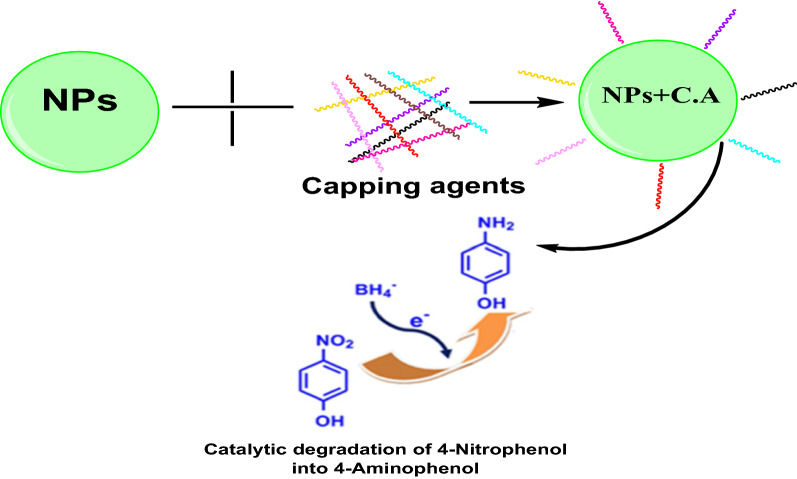
Capped nanoparticles undergoing catalytic degradation of 4-Nitrophenol into 4-Aminophenol

However, researchers have to make sure that nanoparticles should not induce environmental degradation itself while using them in the environmental remediation. Mostly, nanomaterials tend to form aggregates, adsorb into bigger particles or surfaces, or even decomposed [116, 117] that can be toxic to human health and the environment. Meanwhile, the contact of nanomaterials to their environment takes place through their surfaces [118], and hence nanoparticles can even have a higher susceptibility for toxicity. To overcome these limitations and endure their characteristic properties, nanoparticles are generally functionalized, although materials that are used for functionalization have different effects on the physico-chemical properties of nanoparticles [119, 120]. Among them, polymer functionalization of nanoparticles has recently gained significant momentum in the field of environmental remediation due to the integration of both nanoparticles and polymers together under the same system. Polymer-coated nanoparticles are expedient because of their surface properties and pore space, together with their excellent and long-lasting mechanical strength. Moreover, polymer-coated nanoparticles retain their innate features and provide stability and biocompatibility, hence providing a superior surface coating. Furthermore, selectively can also be attained via specific chemical functionalization in order to target certain pollutants. Mainly, due to these properties, the promising use of polymer-coated nanoparticles for remediation and cleaning of environmental pollutants is in practice (Figs. 7 and 8). The basic mechanism of action of polymer-coated nanoparticles comprises of adsorption, catalytic degradation (pollutants), and antibacterial effect especially used in the purification of drinking water [121]. Various types of polymer functionalized nanoparticles used in the environmental remediation are presented in Table 2. These nanomaterials are target specific and do not produce any waste, therefore, there is no requirement to dispose of after treatment, and propose a greener route for environmental remediation [122].

**Table 2:** Polymer capped nanoparticles employed for environmental remediation applications

Nanoparticles	Capping agents	Principle	Substrate	Benefits	References
Ag	Chitosan	Catalysis	Methyl Orange	Efficient and occur in visible light	[89]
Ag	Core–shell magnetic chitosan	Adsorption	Cationic & anionic	Multi-dye adsorption, magnetic separation, and reusability	[90]
Ag	PVA	Biosorption	Manganese ions	Immobilized on *Trichosporon cutaneum R57* strain	[91]
Ag & ZnO	PVP	Photocatalyst	Methylene blue	Efficient as compared to simple ZnO nanoparticles	[123]
Ag	Chitosan	Adsorption	Atrazine (pesticide)	Efficient and reusable	[124]
Ag	Two different chitosan microparticles	Adsorption	Methyl parathion (pesticide)	Efficient and reusable	[125]
Ag & Au	Chitosan	Adsorption	Methyl parathion (pesticide)	Thermostable, highly hydrophilic	[126]
Au	PVP	Adsorption	Mercury	Efficiency can be controlled via optimizing the concentration of PVP	[127]
Fe	PVP	Catalytic adsorption	Bromate	Efficient and prolonged storage	[128]
Au	Chitosan	Sensor	Copper and Zinc ions	Detect even the lowest metal concentration	[129]
Magnetite	Chitosan & polythiophene	Adsorption	Mercury (II)	Efficient and sensitive	[130]
Silver tin sulfide	PEG	Catalysis	Eosin yellow & brilliant green dyes	Efficient, photostable, and recyclable	[131]
TiO_2_	PVA	Photocatalysis	Rhodamine B	Highly efficient, stable, and reusable	[132]
Ag	PVA	Sensor	Hydrogen peroxide	Simple, cost-effective and reliable	[96]

## Conclusions and future implications

In essence, this novel survey unveils the favorable aspects of various capping agents in nanobiotechnology. Although designing the capped nanoparticles exhibiting significantly enhanced biomedical and environmental remediation properties as compared to the uncapped nanoparticles is very challenging, literature is increasing that highlights the unanticipated beneficial role of capping agents in different biological approaches. The controlled size, morphology, and surface composition achieved by nanoparticles’ capping are crucial in determining the vital application of nanoparticles. However, such protocols need to be optimized that could efficaciously validate the capping phenomenon of stabilizers, and more reproducible experiments should be performed that could remove any discrepancies in getting the pure and controlled effect of the appropriate capping agents. Moreover, advanced characterization techniques should be utilized that would finely interpret the role of capping agents in the nanoparticle-stabilizer interface. Besides, in vitro and in vivo toxicity studies of capped nanocomposites should be conducted since the risk assessment of pharmacological and bioremediation activities are requisites to be strictly achieved in laboratory and clinical practices.

## References

[1] Salata O (2004). Applications of nanoparticles in biology and medicine. J Nanobiotechnol.

[2] Wang S, Yang X, Zhou L, Li J, Chen H (2020). 2D nanostructures beyond graphene: preparation, biocompatibility and biodegradation behaviors. J Mater Chem B.

[3] Wang S, Zhou L, Zheng Y, Li L, Wu C, Yang H, Huang M, An X (2019). Synthesis and biocompatibility of two-dimensional biomaterials. Colloids Surf A.

[4] Gulati S, Sachdeva M, Bhasin KK (2018). Capping agents in nanoparticle synthesis: surfactant and solvent system. AIP Conf Proc.

[5] Aisida SO, Batool A, Khan FM, Rahman L, Mahmood A, Ahmad I (2020). Calcination induced PEG-Ni-ZnO nanorod composite and its biomedical applications. Mater Chem Phys.

[6] Aisida SO, Alnasir MH, Botha S, Bashir AKH, Bucher R, Ahmad I (2020). The role of polyethylene glycol on the microstructural, magnetic and specific absorption rate in thermoablation properties of Mn-Zn ferrite nanoparticles by sol–gel protocol. Eur Polymer J.

[7] Aisida SO, Ugwoke E, Uwais A, Iroegbu C, Botha S, Ahmad I (2019). Incubation period induced biogenic synthesis of PEG enhanced Moringa oleifera silver nanocapsules and its antibacterial activity. J Polym Res.

[8] Rajendran K, Sen S (2015). Effect of capping agent on antimicrobial activity of nanoparticles. Der Pharmacia Lettre..

[9] Javed R, Usman M, Tabassum S, Zia M (2016). Effect of capping agents: structural, optical and biological properties of ZnO nanoparticles. Appl Surf Sci.

[10] Anderson SD, Gwenin VV, Gwenin CD (2019). Magnetic functionalized nanoparticles for biomedical, drug delivery and imaging applications. Nanoscale Res Lett.

[11] Römer I, Briffa SM, Dasilva YAR, Hapiuk D, Trouillet V, Palmer RE (2019). Impact of particle size, oxidation state and capping agent of different cerium dioxide nanoparticles on the phosphate-induced transformations at different pH and concentration. PLoS ONE.

[12] Parveen K, Banse V, Ledwani L (2016). Green synthesis of nanoparticles: their advantages and disadvantages. AIP Conf Proc.

[13] Niu Z, Li Y (2014). Removal and utilization of capping agents in nanocatalysis. Chem Mater.

[14] Gulati S, Sachdeva M, Bhasin KK (2018). Capping agents in nanoparticle synthesis: surfactant and solvent system. AIP Conf Proc.

[15] Radini IA, Hasan N, Malik MA, Khan Z (2018). Biosynthesis of iron nanoparticles using Trigonella foenum-graecum seed extract for photocatalytic methyl orange dye degradation and antibacterial applications. J Photochem Photobiol B.

[16] Shameli K, Ahmad MB, Zamanian A, Sangpour P, Shabanzadeh P, Abdollahi Y (2012). Green biosynthesis of silver nanoparticles using Curcuma longa tuber powder. Int J Nanomed.

[17] Rajitha B, Malla RR, Vadde R, Kasa P, Prasad GLV, Farran B, et al. Horizons of nanotechnology applications in female specific cancers. Seminars in Cancer Biology. Academic Press; 2019. 10.1016/j.semcancer.2019.07.005 31301361

[18] Alberto Zamora-Justo J, Abrica-González P, Rocael Vázquez-Martínez G, Muñoz-Diosdado A, Abraham Balderas-López J, Ibáñez-Hernández M (2019). Polyethylene glycol-coated gold nanoparticles as DNA and atorvastatin delivery systems and cytotoxicity evaluation. J Nanomateria.

[19] Pinzaru I, Coricovac D, Dehelean C, Moacă EA, Mioc M, Baderca F (2018). Stable PEG-coated silver nanoparticles—a comprehensive toxicological profile. Food Chem Toxicol.

[20] Singletary M, Hagerty S, Muramoto S, Daniels Y, MacCrehan WA, Stan G (2017). PEGylation of zinc nanoparticles amplifies their ability to enhance olfactory responses to odorant. PLOS ONE..

[21] Teodorescu M, Bercea M (2015). Poly(vinylpyrrolidone)—a versatile polymer for biomedical and beyond medical applications. Polym Plast Technol Eng.

[22] Okoroh DO, Ozuomba JO, Aisida SO, Asogwa PU (2019). Properties of zinc ferrite nanoparticles due to PVP mediation and annealing at 500 °C. Adv. Nanoparticles..

[23] Goodarz Naseri M, Saion E, Khalil Zadeh N (2013). The amazing effects and role of PVP on the crystallinity, phase composition and morphology of nickel ferrite nanoparticles prepared by thermal treatment method. Int Nano Lett.

[24] Pandey G, Singh S, Hitkari G (2018). Synthesis and characterization of polyvinyl pyrrolidone (PVP)-coated Fe3O4 nanoparticles by chemical co-precipitation method and removal of Congo red dye by adsorption process. Int Nano Lett.

[25] Ahlberg S, Antonopulos A, Diendorf J, Dringen R, Epple M, Flöck R (2014). PVP-coated, negatively charged silver nanoparticles: a multi-center study of their physicochemical characteristics, cell culture and in vivo experiments. Beilstein J Nanotechnol.

[26] Junaidi J, Triyana K, Suharyadi E, Sabarman H, Wu L (2017). The roles of polyvinyl alcohol (PVA) as the capping agent on the polyol method for synthesizing silver nanowires. J Nano Res.

[27] Aisida SO, Ahmad I, Zhao T-K, Maaza M, Ezema FI (2020). Calcination effect on the photoluminescence, optical, structural, and magnetic properties of polyvinyl alcohol doped ZnFe2O4 nanoparticles. J Macromol Sci Part B.

[28] Kyrychenko A, Pasko DA, Kalugin ON (2017). Poly(vinyl alcohol) as a water protecting agent for silver nanoparticles: the role of polymer size and structure. Phys Chem Chem Phys.

[29] Gaaz TS, Sulong AB, Akhtar MN, Kadhum AAH, Mohamad AB, Al-Amiery AA (2015). Properties and applications of polyvinyl alcohol, halloysite nanotubes and their nanocomposites. Molecules.

[30] Kundu TK, Karak N, Barik P, Saha S (2011). Optical properties of ZnO nanoparticles prepared by chemical method using poly (vinylalcohol) (PVA) as capping agent. IJSCE..

[31] Budi L, Rahayu H, Wulandari IO, Santjojo DH, Sabarudin A (2017). Synthesis and characterization of Fe_3_O_4_ nanoparticles using polyvinyl alcohol (PVA) as capping agent and glutaraldehyde (GA) as crosslinker. IOP Conf Ser Mater Sci Eng.

[32] Au L, Lim B, Colletti P, Jun YS, Xia Y (2010). Synthesis of gold microplates using bovine serum albumin as a reductant and a stabilizer. Chemistry.

[33] Bolaños K, Kogan MJ, Araya E (2019). Capping gold nanoparticles with albumin to improve their biomedical properties. Int J Nanomed.

[34] JyothiKumar A, Kailasnath M, Simon J, Mathew S (2019). BSA stabilized gold nanoparticles: synthesis and characterization. Mater Today Proc.

[35] Li H, Yang X (2015). Bovine serum albumin-capped CdS quantum dots as an inner-filter effect sensor for rapid detection and quantification of protamine and heparin. Anal Methods.

[36] Zhao S, Gao Y, Tan J, Zhu Y, Ying X, Zhang M (2019). Facile synthesis and antibacterial applications of cuprous oxide/bovine serum albumin hierarchical nanocomposite particles. SN Appl Sci.

[37] Resende JE, Gonçalves MA, Oliveira LCA, Da Cunha EFF, Ramalho TC (2014). Use of ethylenediaminetetraacetic acid as a scavenger for chromium from “‘wet blue’” leather waste: thermodynamic and kinetics parameters. J Chem.

[38] Harish GS, Reddy PS (2013). Synthesis and Characterization of water soluble ZnS: Ce, Cu co-doped Nanoparticles: Effect of EDTA Concentration. Int Jo Sci Res.

[39] Reddy DA, Murali G, Vijayalakshmi RP, Reddy BK (2011). Room-temperature ferromagnetism in EDTA capped Cr-doped ZnS nanoparticles. Appl Phys A Mater Sci Process.

[40] Rahal HT, Awad R, Abdel-Gaber AM, El-Said Bakeer D (2017). Synthesis, characterization, and magnetic properties of pure and EDTA-capped NiO nanosized particles. J Nanomater.

[41] Yi Y, Zhang Y, Wang Y, Shen L, Jia M, Huang Y (2014). Ethylenediaminetetraacetic acid as capping ligands for highly water-dispersible iron oxide particles. Nanoscale Res Lett.

[42] Elgadir A, Uddin M, Ferdosh S, Adam S, Jalal A, Chowdhury AJK (2014). Impact of chitosan composites and chitosan nanoparticle composites on various drug delivery systems: a review. J Food Drug Anal.

[43] Mohan CO, Ravishankar CN, Lalitha KV, Srinivasa Gopal TK (2012). Effect of chitosan edible coating on the quality of double filleted Indian oil sardine (Sardinella longiceps) during chilled storage. Food Hydrocolloids.

[44] Pang Y, Qin A, Lin X, Yang L, Wang Q, Wang Z (2017). Biodegradable and biocompatible high elastic chitosan scaffold is cell-friendly both in vitro and in vivo. Oncotarget..

[45] Deng HH, Lin XL, Liu YH, Li KL, Zhuang QQ, Peng HP (2017). Chitosan-stabilized platinum nanoparticles as effective oxidase mimics for colorimetric detection of acid phosphatase. Nanoscale..

[46] Franconetti A, Carnerero JM, Prado-Gotor R, Cabrera-Escribano F, Jaime C (2019). Chitosan as a capping agent: insights on the stabilization of gold nanoparticles. Carbohyd Polym.

[47] Cinteza LO, Scomoroscenco C, Voicu SN, Nistor CL, Nitu SG, Trica B (2018). Chitosan-stabilized ag nanoparticles with superior biocompatibility and their synergistic antibacterial effect in mixtures with essential oils. Nanomaterials..

[48] Jayaramudu T, Varaprasad K, Pyarasani RD, Reddy KK, Kumar KD, Akbari-Fakhrabadi A (2019). Chitosan capped copper oxide/copper nanoparticles encapsulated microbial resistant nanocomposite films. Int J Biol Macromol.

[49] Mohan CO, Gunasekaran S, Ravishankar CN (2019). Chitosan-capped gold nanoparticles for indicating temperature abuse in frozen stored products. NPJ Sci Food.

[50] Aisida SO, Ugwu K, Akpa PA, Nwanya AC, Ejikeme PM, Botha S (2019). Biogenic synthesis and antibacterial activity of controlled silver nanoparticles using an extract of Gongronema Latifolium. Mater Chem Phys.

[51] Aisida SO, Madubuonu N, Alnasir MH, Ahmad I, Botha S, Maaza M (2020). Biogenic synthesis of iron oxide nanorods using Moringa oleifera leaf extract for antibacterial applications. Appl Nanosci..

[52] Aisida SO, Ugwu K, Akpa PA, Nwanya AC, Nwankwo U, Botha SS (2019). Biosynthesis of silver nanoparticles using bitter leave (Veronica amygdalina) for antibacterial activities. Surf Interfaces..

[53] Dos Santos Corrêa A, Contreras LA, Keijok WJ, Barcelos DHF, Pereira ACH, Kitagawa RR (2018). Virola oleifera-capped gold nanoparticles showing radical-scavenging activity and low cytotoxicity. Mater Sci Eng C Mater Biol Appl..

[54] Rashid S, Azeem M, Khan SA, Shah MM, Ahmad R (2019). Characterization and synergistic antibacterial potential of green synthesized silver nanoparticles using aqueous root extracts of important medicinal plants of Pakistan. Colloids Surf, B.

[55] Ramanathan S, Gopinath SCB, Anbu P, Lakshmipriya T, Kasim FH, Lee C-G (2018). Eco-friendly synthesis of Solanum trilobatum extract-capped silver nanoparticles is compatible with good antimicrobial activities. J Mol Struct.

[56] Madubuonu N, Aisida SO, Ali A, Ahmad I, Zhao T, Botha S (2019). Biosynthesis of iron oxide nanoparticles via a composite of Psidium guavaja-Moringa oleifera and their antibacterial and photocatalytic study. J Photochem Photobiol, B.

[57] Ezhilarasi AA, Vijaya JJ, Kennedy LJ, Kaviyarasu K (2020). Green mediated NiO nano-rods using Phoenix dactylifera (Dates) extract for biomedical and environmental applications. Mater Chem Phys.

[58] Rasheed T, Bilal M, Iqbal HMN, Li C (2017). Green biosynthesis of silver nanoparticles using leaves extract of Artemisia vulgaris and their potential biomedical applications. Colloids Surf B Biointerfaces..

[59] Pandiyan N, Murugesan B, Arumugam M, Sonamuthu J, Samayanan S, Mahalingam S (2019). Ionic liquid - A greener templating agent with Justicia adhatoda plant extract assisted green synthesis of morphologically improved Ag-Au/ZnO nanostructure and it’s antibacterial and anticancer activities. J Photochem Photobiol, B.

[60] Senthilkumar N, Nandhakumar E, Priya P, Soni D, Vimalan M, Potheher IV (2017). Synthesis of ZnO nanoparticles using leaf extract of *Tectona grandis* (L.) and their anti-bacterial, anti-arthritic, anti-oxidant and in vitro cytotoxicity activities. New J Chem..

[61] Naz S, Islam M, Tabassum S, Fernandes NF, Carcache de Blanco EJ, Zia M (2019). Green synthesis of hematite (α-Fe2O3) nanoparticles using Rhus punjabensis extract and their biomedical prospect in pathogenic diseases and cancer. J Mol Struct.

[62] Satpathy S, Patra A, Ahirwar B, Hussain MD (2020). Process optimization for green synthesis of gold nanoparticles mediated by extract of Hygrophila spinosa T. Anders and their biological applications. Phys E Low Dimens Syst Nanostruct.

[63] Yadav R, Saini H, Kumar D, Pasi S, Agrawal V (2019). Bioengineering of *Piper longum* L. extract mediated silver nanoparticles and their potential biomedical applications. Mater Sci Eng C..

[64] Hola K, Markova Z, Zoppellaro G, Tucek J, Zboril R (2015). Tailored functionalization of iron oxide nanoparticles for MRI, drug delivery, magnetic separation and immobilization of biosubstances. Biotechnol Adv.

[65] Cao S-W, Zhu Y-J, Ma M-Y, Li L, Zhang L (2008). Hierarchically Nanostructured Magnetic Hollow Spheres of Fe3O4 and γ-Fe2O3: preparation and Potential Application in Drug Delivery. J Phys Chem C.

[66] Yang X, Chen L, Han B, Yang X, Duan H (2010). Preparation of magnetite and tumor dual-targeting hollow polymer microspheres with pH-sensitivity for anticancer drug-carriers. Polymer.

[67] Mirza AU, Kareem A, Nami SAA, Khan MS, Rehman S, Bhat SA (2018). Biogenic synthesis of iron oxide nanoparticles using *Agrewia optiva* and *Prunus persica* phyto species: characterization, antibacterial and antioxidant activity. J Photochem Photobiol, B.

[68] Shalaby TI, El-Refaie WM (2018). Bioadhesive chitosan-coated cationic nanoliposomes with improved insulin encapsulation and prolonged oral hypoglycemic effect in diabetic mice. J Pharm Sci.

[69] Ravichandran V, Vasanthi S, Shalini S, Shah SAA, Tripathy M, Paliwal N (2019). Green synthesis, characterization, antibacterial, antioxidant and photocatalytic activity of Parkia speciosa leaves extract mediated silver nanoparticles. Results Phys.

[70] Chandran S, Ravichandran V, Chandran S, Chemmanda J, Chandarshekar B (2016). Biosynthesis of PVA encapsulated silver nanoparticles. J Appl Res Technol..

[71] Devanand Venkatasubbu G, Ramasamy S, Ramakrishnan V, Kumar J (2013). Folate targeted PEGylated titanium dioxide nanoparticles as a nanocarrier for targeted paclitaxel drug delivery. Adv Powder Technol.

[72] Javed R, Rais F, Fatima H, ul Haq I, Kaleem M, Naz SS (2020). Chitosan encapsulated ZnO nanocomposites: fabrication, characterization, and functionalization of bio-dental approaches. Mater Sci Eng C..

[73] Prabha G, Raj V (2016). Preparation and characterization of polymer nanocomposites coated magnetic nanoparticles for drug delivery applications. J Magn Magn Mater.

[74] Bharathi D, Ranjithkumar R, Vasantharaj S, Chandarshekar B, Bhuvaneshwari V (2019). Synthesis and characterization of chitosan/iron oxide nanocomposite for biomedical applications. Int J Biol Macromol.

[75] Popov AL, Han B, Ermakov AM, Savintseva IV, Ermakova ON, Popova NR (2020). PVP-stabilized tungsten oxide nanoparticles: pH sensitive anti-cancer platform with high cytotoxicity. Mater Sci Eng, C.

[76] Selvam R, Ramasamy S, Mohiyuddin S, Enoch IVMV, Gopinath P, Filimonov D (2018). Molecular encapsulator–appended poly(vinyl alcohol) shroud on ferrite nanoparticles. Augmented cancer–drug loading and anticancer property. Mater Sci Eng C..

[77] Lubambo AF, Ono L, Drago V, Mattoso N, Varalda J, Sierakowski M-R (2015). Tuning Fe3O4 nanoparticle dispersion through pH in PVA/guar gum/electrospun membranes. Carbohydr Polym.

[78] Ashfaq M, Khan S, Verma N (2014). Synthesis of PVA-CAP-based biomaterial in situ dispersed with Cu nanoparticles and carbon micro-nanofibers for antibiotic drug delivery applications. Biochem Eng J.

[79] Ngernpimai S, Thomas C, Maensiri S, Siri S (2012). Stability and cytotoxicity of well-dispersed magnetite nanoparticles prepared by hydrothermal method. Adv Mater Res.

[80] Pinto RJB, Marques PAAP, Neto CP, Trindade T, Daina S, Sadocco P (2009). Antibacterial activity of nanocomposites of silver and bacterial or vegetable cellulosic fibers. Acta Biomater.

[81] Trandafilović LV, Božanić DK, Dimitrijević-Branković S, Luyt AS, Djoković V (2012). Fabrication and antibacterial properties of ZnO–alginate nanocomposites. Carbohyd Polym.

[82] Erazo A, Mosquera SA, Rodríguez-Paéz JE (2019). Synthesis of ZnO nanoparticles with different morphology: study of their antifungal effect on strains of *Aspergillus niger* and *Botrytis cinerea*. Mater Chem Phys.

[83] Ferreira FV, Mariano M, Lepesqueur LSS, Pinheiro IF, Santos LG, Burga-Sánchez J (2019). Silver nanoparticles coated with dodecanethiol used as fillers in non-cytotoxic and antifungal PBAT surface based on nanocomposites. Mater Sci Eng, C.

[84] Naz S, Akhtar J, Chaudhary MF, Zia M (2018). Low-temperature synthesis of hierarchical structures of copper oxide and their superior biological activity. IET Nanobiotechnol.

[85] Hajipour MJ, Fromm KM, Akbar Ashkarran A, Jimenez de Aberasturi D, de Larramendi IR, Rojo T (2012). Antibacterial properties of nanoparticles. Trends Biotechnol.

[86] Fresta M, Puglisi G, Giammona G, Cavallaro G, Micali N, Furneri PM (1995). Pefloxacine mesilate- and ofloxacin-loaded polyethylcyanoacrylate nanoparticles: characterization of the colloidal drug carrier formulation. J Pharm Sci.

[87] Ali JS, Mannan A, Nasrullah M, Ishtiaq H, Naz S, Zia M (2020). Antimicrobial, antioxidative, and cytotoxic properties of Monotheca buxifolia assisted synthesized metal and metal oxide nanoparticles. Inorganic Nano-Metal Chem.

[88] Sondi I, Salopek-Sondi B (2004). Silver nanoparticles as antimicrobial agent: a case study on *E. coli* as a model for Gram-negative bacteria. J Colloid Interface Sci.

[89] Nithya A, JeevaKumari HL, Rokesh K, Ruckmani K, Jeganathan K, Jothivenkatachalam K (2015). A versatile effect of chitosan-silver nanocomposite for surface plasmonic photocatalytic and antibacterial activity. J Photochem Photobiol, B.

[90] Ramalingam B, Khan MdMR, Mondal B, Mandal AB, Das SK (2015). Facile synthesis of silver nanoparticles decorated magnetic-chitosan microsphere for efficient removal of dyes and microbial contaminants. ACS Sustain Chem Eng..

[91] Bryaskova R, Georgieva N, Pencheva D, Todorova Z, Lazarova N, Kantardjiev T (2014). Synthesis and characterization of hybrid materials with embedded silver nanoparticles and their application as antimicrobial matrices for waste water purification. Colloids Surf, A.

[92] Gélvez APC, Farias LHS, Pereira VS, da Silva ICM, Costa AC, Dias CGBT (2018). Biosynthesis, characterization and leishmanicidal activity of a biocomposite containing AgNPs-PVP-glucantime. Nanomedicine..

[93] Kvítek L, Panáček A, Soukupová J, Kolář M, Večeřová R, Prucek R (2008). Effect of surfactants and polymers on stability and antibacterial activity of silver nanoparticles (NPs). J Phys Chem C.

[94] Jia Z, Sun H, Gu Q (2013). Preparation of Ag nanoparticles with triethanolamine as reducing agent and their antibacterial property. Colloids Surf A.

[95] Javed R, Ahmed M, Haq IU, Nisa S, Zia M (2017). PVP and PEG doped CuO nanoparticles are more biologically active: antibacterial, antioxidant, antidiabetic and cytotoxic perspective. Mater Sci Eng C Mater Biol Appl..

[96] Ajitha B, Reddy YAK, Reddy PS, Jeon H-J, Ahn CW (2016). Role of capping agents in controlling silver nanoparticles size, antibacterial activity and potential application as optical hydrogen peroxide sensor. RSC Adv..

[97] Martínez-Castañón GA, Niño-Martínez N, Martínez-Gutierrez F, Martínez-Mendoza JR, Ruiz F (2008). Synthesis and antibacterial activity of silver nanoparticles with different sizes. J Nanopart Res.

[98] Dhillon GS, Kaur S, Brar SK (2014). Facile fabrication and characterization of chitosan-based zinc oxide nanoparticles and evaluation of their antimicrobial and antibiofilm activity. Int Nano Lett..

[99] Stefan M, Melnig V, Pricop D, Neagu A, Mihasan M, Tartau L (2013). Attenuated effects of chitosan-capped gold nanoparticles on LPS-induced toxicity in laboratory rats. Mater Sci Eng C Mater Biol Appl..

[100] Kim JS, Kuk E, Yu KN, Kim J-H, Park SJ, Lee HJ (2007). Antimicrobial effects of silver nanoparticles. Nanomed Nanotechnol Biol Med.

[101] Morones JR, Elechiguerra JL, Camacho A, Holt K, Kouri JB, Ramírez JT (2005). The bactericidal effect of silver nanoparticles. Nanotechnology..

[102] Milanezi FG, Meireles LM, de Christo Scherer MM, de Oliveira JP, da Silva AR, de Araujo ML, Scherer R (2019). Antioxidant, antimicrobial and cytotoxic activities of gold nanoparticles capped with quercetin. Saudi Pharm J.

[103] Zhai X, Zhang C, Zhao G, Stoll S, Ren F, Leng X (2017). Antioxidant capacities of the selenium nanoparticles stabilized by chitosan. J Nanobiotechnol.

[104] Matos AI, Carreira B, Peres C, Moura LIF, Conniot J, Fourniols T (2019). Nanotechnology is an important strategy for combinational innovative chemo-immunotherapies against colorectal cancer. J Control Release.

[105] Muhammad Z, Raza A, Ghafoor S, Naeem A, Naz SS, Riaz S (2016). PEG capped methotrexate silver nanoparticles for efficient anticancer activity and biocompatibility. Eur J Pharm Sci.

[106] Alfaro A, León A, Guajardo-Correa E, Reúquen P, Torres F, Mery M (2019). MgO nanoparticles coated with polyethylene glycol as carrier for 2-Methoxyestradiol anticancer drug. PLOS ONE.

[107] Schleich N, Sibret P, Danhier P, Ucakar B, Laurent S, Muller RN (2013). Dual anticancer drug/superparamagnetic iron oxide-loaded PLGA-based nanoparticles for cancer therapy and magnetic resonance imaging. Int J Pharm.

[108] Agrawal AK, Urimi D, Harde H, Kushwah V, Jain S (2015). Folate appended chitosan nanoparticles augment the stability, bioavailability and efficacy of insulin in diabetic rats following oral administration. RSC Adv.

[109] Wu Z-H, Ping Q-N, Song Y-M, Lei X-M, Li J-Y, Cai P (2004). Studies on the insulin-liposomes double-coated by chitosan and chitosan-EDTA conjugates. Acta Pharmaceutica Sinica..

[110] Masciangioli T, Zhang W-X (2003). Peer Reviewed: environmental technologies at the nanoscale. Environ Sci Technol.

[111] Boubel RW, Fox DL, Turner DB, Stern AC. The history of air pollution. Fundam Air Pollut 1994;3–18.

[112] Saradha B, Mathur PP (2006). Effect of environmental contaminants on male reproduction. Environ Toxicol Pharmacol.

[113] Rajan CSR. Nanotechnology in groundwater remediation. IJESD. 2011;182–7.

[114] Lövestam G, Rauscher H, Roebben G, Klüttgen BS, Gibson N, Putaud JP, Stamm H. Considerations on a definition of nanomaterial for regulatory purposes. Joint Research Centre (JRC) Reference Reports. 2010;80:00–41.

[115] Nurmi JT, Tratnyek PG, Sarathy V, Baer DR, Amonette JE, Pecher K (2005). Characterization and properties of metallic iron nanoparticles: spectroscopy, electrochemistry, and kinetics. Environ Sci Technol.

[116] Bhatt I, Tripathi BN (2011). Interaction of engineered nanoparticles with various components of the environment and possible strategies for their risk assessment. Chemosphere.

[117] Klaine SJ, Alvarez PJJ, Batley GE, Fernandes TF, Handy RD, Lyon DY (2008). Nanomaterials in the environment: behavior, fate, bioavailability, and effects. Environ Toxicol Chem.

[118] Moyano DF, Rotello VM (2011). Nano meets biology: structure and function at the nanoparticle interface. Langmuir.

[119] Liu J, Legros S, Ma G, Veinot JGC, von der Kammer F, Hofmann T (2012). Influence of surface functionalization and particle size on the aggregation kinetics of engineered nanoparticles. Chemosphere.

[120] Lu A-H, Salabas EL, Schüth F (2007). Magnetic nanoparticles: synthesis, protection, functionalization, and application. Angew Chem.

[121] Potara M, Focsan M, Craciun A-M, Botiz I, Astilean S. 15-Polymer-coated plasmonic nanoparticles for environmental remediation: Synthesis, functionalization, and properties. In: Hussain CM, Mishra AK, editors. New polymer nanocomposites for environmental remediation; 2018, p. 361–87.

[122] Aashima, Mehta SK. Chapter 18 - Impact of functionalized nanomaterials towards the environmental remediation: challenges and future needs. In: Mustansar Hussain C, editor. Handbook of functionalized nanomaterials for industrial applications; 2020, p. 505–24.

[123] Khademalrasool M, Farbod M, Iraji zad A (2016). Preparation of ZnO nanoparticles/Ag nanowires nanocomposites as plasmonic photocatalysts and investigation of the effect of concentration and diameter size of Ag nanowires on their photocatalytic performance. J Alloys Compounds..

[124] Saifuddin NM, Nian CY, Zhan L, Ning K (2011). Chitosan-silver nanoparticles composite as point-of-use drinking water filtration system for household to remove pesticides in water. Asian J Biochem.

[125] Yoshizuka K, Lou Z, Inoue K (2000). Silver-complexed chitosan microparticles for pesticide removal. React Funct Polym.

[126] Dwivedi C, Gupta A, Chaudhary A, Nandi CK (2014). Gold nanoparticle chitosan composite hydrogel beads show efficient removal of methyl parathion from waste water. RSC Adv..

[127] Kamarudin KS, Mohamad MF, Fathilah NN, Mohamed MS (2010). Effects of PVP concentration on the formation of size and shape of gold (Au) nanoparticles for mercury adsorption. J Appl Sci.

[128] Xu X, Wang Q, Choi HC, Kim YH (2010). Encapsulation of iron nanoparticles with PVP nanofibrous membranes to maintain their catalytic activity. J Membr Sci.

[129] Sugunan A, Thanachayanont C, Dutta J, Hilborn JG (2005). Heavy-metal ion sensors using chitosan-capped gold nanoparticles. Sci Technol Adv Mater.

[130] Morsi RE, Al-Sabagh AM, Moustafa YM, ElKholy SG, Sayed MS (2018). Polythiophene modified chitosan/magnetite nanocomposites for heavy metals and selective mercury removal. Egypt J Petrol.

[131] Shambharkar BH, Chowdhury AP (2016). Ethylene glycol mediated synthesis of Ag8SnS6 nanoparticles and their exploitation in the degradation of eosin yellow and brilliant green. RSC Adv..

[132] Arularasu MV (2019). Effect of organic capping agents on the optical and photocatalytic activity of mesoporous TiO2 nanoparticles by sol–gel method. SN Appl Sci..

